# Incidence of menopausal symptoms in postmenopausal breast cancer patients treated with aromatase inhibitors

**DOI:** 10.18632/oncotarget.17194

**Published:** 2017-04-18

**Authors:** Dongsheng Hong, Ling Bi, Jun Zhou, Yinghui Tong, Qingwei Zhao, Jing Chen, Xiaoyang Lu

**Affiliations:** ^1^ Department of Pharmacy, The First Affiliated Hospital of College of Medicine, Zhejiang University, Hangzhou, 310003, P.R. China; ^2^ Department of Stomatology, The First Affiliated Hospital, School of Medicine, Zhejiang University, Hangzhou, 310003, P.R. China; ^3^ Department of Pharmacy, Zhejiang Cancer Hospital, Hangzhou, 310022, P.R. China; ^4^ Department of Medical Oncology, The First Affiliated Hospital of College of Medicine, Zhejiang University, Hangzhou, 310003, P.R. China

**Keywords:** breast cancer, aromatase inhibitors, menopausal symptoms, hot flashes, meta-analysis

## Abstract

Aromatase inhibitors (AIs) are the standard of care for postmenopausal women with estrogen receptor-positive breast cancer. Here, we performed a meta-analysis to evaluate the occurrence of menopausal symptoms in breast cancer patients receiving the AI therapy. Patients treated with AIs had an increased risk of all-grade arthralgia (1.63 [95% CI: 1.34–1.98]) and insomnia (1.24 [95% CI: 1.14–1.34]). The overall incidence of hot flashes, fatigue, arthralgia, sweating, and insomnia in patients receiving AIs was 30.47% (95% CI: 25.51%–35.93%), 17.16% (95% CI: 14%–20.85%), 17.91% (95% CI: 11.29%–27.22%), 14.64% (95% CI: 11.46%–18.52%), and 16.52% (95% CI: 12.45%–21.6 %), respectively. Both arthralgia (RR = 0.34, 95% CI: 0.16–0.75) and sweating (RR = 11.02, 95% CI: 4.11–29.57) differed between patients with early- and advanced-stage breast cancer. Our findings indicates that AIs are associated with a significant risk of developing arthralgia and insomnia in breast cancer patients. Effective early detection and management of menopausal symptoms would likely lead to safer use of AIs in breast cancer patients.

## INTRODUCTION

Breast cancer is one of the most common malignancies and causes of tumor-related deaths among women worldwide [1]. Most of the breast cancer patients are postmenopausal at the time of diagnosis, or reach menopause following anti-cancer treatment. Aromatase inhibitors (AIs) are a cornerstone of the standard of care for most postmenopausal breast cancer patients who are progesterone receptor and/or estrogen-receptor positive [2]. Anastrozole, letrozole, and exemestane are AIs that have been used in randomized control trials (RCTs) and demonstrated advantage compared with tamoxifen [3–5]. Several studies have indicated that AIs may have side effects, such as genitourinary or musculoskeletal discomfort [6, 7]. However, the full impact of AIs on menopausal symptoms (MS) in breast cancer patients is not known.

MS related to breast cancer include hot flashes, fatigue, arthralgia, sweating, and insomnia; more than 50% of menopausal women report sweating and hot flashes [8–10]. MS induced by AIs have been reported in several RCTs, but the specific risk of MS associated with AIs has not been defined. Here, we conducted a systematic review and meta-analysis to evaluate the association of AI therapies with MS in breast cancer patients.

## RESULTS

### Search results and study characteristics

**Figure 1:**
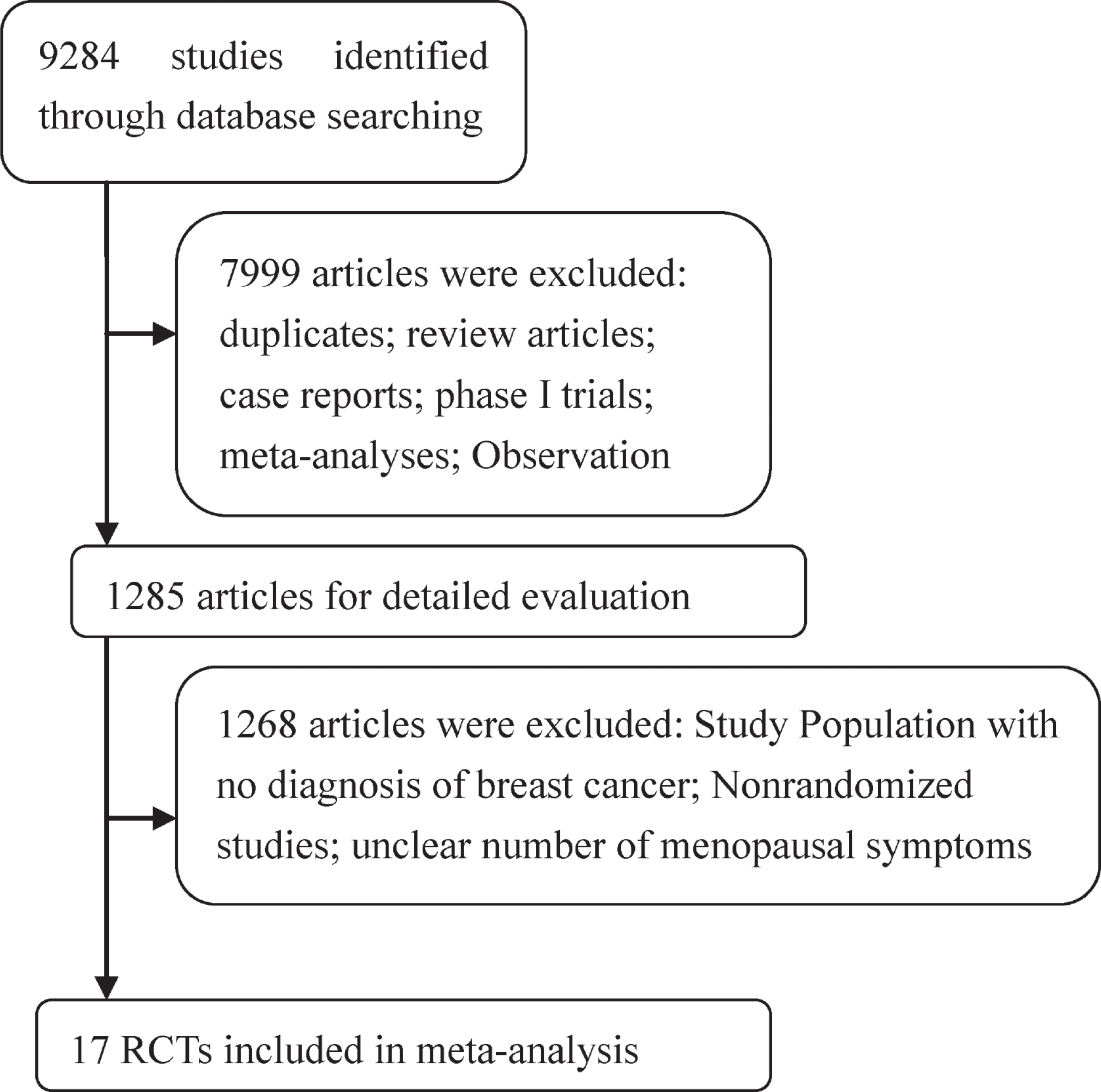
Flow chart demonstrating process of study selection

Through initial search, 9,284 potentially relevant studies were identified. After reviewing titles and abstracts, 1,285 studies were selected for full evaluation. Ultimately, 17 studies met our inclusion criteria, and 9,054 subjects were included in our analysis [5, 11–26]. Figure 1 outlines the selection process in detail. From the selected studies, 14 RCTs were based in Europe [5, 11–23], seven in North America [12, 14, 15, 18, 19, 23, 24], seven in the Asia-Pacific region [12–15, 19, 25, 26], and one in Africa [12]. The age of subjects ranged from 35 to 96 years. Duration of follow-up times ranged from 18 to 100 months, but the majority had 30-month follow-up times. The quality of the 17 studies was high: five studies had Jadad scores of 5 [13, 15, 17–19], six studies had Jadad scores of 4 [5, 16, 20–22, 26], and six studies had Jadad scores of 3 [11, 12, 14, 23–25]. The detailed information is shown in Tables 1 and 4, and the Supplementary Material is shown in Supplementary Table 3.

**Table 1:** Characteristics of the 17 randomized clinical trials included in our study

	Aromatase Inhibitors	Tamoxifen/Placebo
Eligible studies		
No.unique studies	17	11
Duration of follow-up, median (IQR), months	36.95 (30.23–54.53)	37.9 (30.6–55.7)
Patients		
Total	25062	20539
Median (IQR), No.	445 (230–2770)	2338 (349–3093)
Age, median (IQR), years	64 (62.5–64.2)	63.2 (60.9–64)
Location		
Europe	14	10
North America	7	3
Asia-Pacific	7	4
Africa	1	0

Abbreviation: IQR, interquartile range.

**Table 2:** Incidence of menopausal symptoms in postmenopausal breast cancer patients assigned to aromatase inhibitor intervention

Menopausal Symptoms	Number of Included Studies	Number of Menopausal Symptoms	Number of Patients	Incidence (95% CI)	Heterogeneity,Q; P; I^2^
Hot flashes	14	7870	19749	30.47% (25.51%–35.93%)	0.1982; < 0.0001; 98.3%
Fatigue	11	2331	12175	17.16% (14%–20.85%)	0.1136; < 0.0001; 94.3%
Arthralgia	11	1672	12421	17.91% (11.29%–27.22%)	0.797; < 0.0001; 98.8%
Sweating	4	1209	7392	14.64% (11.46%–18.52%)	0.0631; < 0.0001; 93.4%
Insomnia	5	1640	10013	16.52% (12.45%–21.60%)	0.1223; < 0.0001; 96.6%

**Table 3:** Meta-analysis of other menopausal symptoms in postmenopausal breast cancer patients assigned to aromatase inhibitor or control intervention

Menopausal Symptoms	Number of Included Studies	Number of Menopausal Symptoms		OR (95%CI)	*P*-value	Heterogeneity,Q; P; I^2^
		Aromatase inhibitor	Control			
Hot flashes	9	5691/15467	6075/15500	0.9071 (0.8086–1.0177)	0.0966	0.0211; < 0.0001; 79.8%
Fatigue	6	2260/11454	2201/11484	1.0389 (0.9728–1.1094)	0.2552	0; 0.9753; 0%
Sweating	4	1205/7130	1247/7165	0.9626 (0.8146–1.1375)	0.4284	0.0158; 0.0254; 72.8%

OR = odds ratio.

**Table 4:** Baseline characteristics of randomized clinical trials included in the meta-analysis

Author(Publication Date)	Trial phase	Tumor staging	Interventions	Age (range, years)	follow-up (media,months)	Events of Menopausal Symptoms					No. of Patiens	Jadadscore	References Number
						Hot Flashes	Fatigue	Arthralgia	Sweating	Insomnia			
Tryfonidis (2016)	Phase 2 Trial	Advanced-stage breast cancer	Anastrozole (1 mg/d)	63.9 (42.8–84.4)	18	13	21	19	NR	NR	35	3	11
Richard (2015)	Phase 3 trial	Advanced-stage breast cancer	Letrozole 2.5 mg/d	64 (56–70)	29.6	9	18	12	NR	NR	77	3	12
Stephen (2013)	Phase 3 trial	Advanced-stage breast cancer	Fulvestrant plus anastrozole	63.8 (57–72)	37.9	88	7	97	NR	75	241	5	13
			Fulvestrant plus placebo	63.4 (57–73.5)	NR	81	8	98	NR	63	230		
Hiroji (2013)	Phase 3 trial	Advanced-stage breast cancer	Anastrozole (1 mg/d)	t64 (9)*	60.1	22	2	25	NR	NR	149	3	25
Paul (2013)	Phase 3 trial	Early-stage breast cancer	Anastrozole	64.3	49.2	2101	NR	231	NR	NR	3759	3	24
Baselga (2012)	Phase 3 trial	Advanced-stage breast cancer	Exemestane	62	NR	NR	26	16	NR	8	238	3	14
Velde (2011)	Phase 3 trial	Early-stage breast cancer	Exemestane (25 mg/d)	64 (35–96)	61.2	NR	NR	NR	NR	654	4852	5	15
			exemestane following tamoxifen	NR	NR	NR	NR	NR	NR	504	4814		
Tomohiko (2010)	Phase 3 trial	I, IIA, IIB/IIIA/IIIB	Anastrozole (1 mg/d)	59.5 (7.4)*	42	126	92	175	NR	NR	347	4	26
			Tamoxifen (20 mg/d)	60.2 (7.4)*	NR	156	89	111	NR	NR	349		
Arimidex (2008)	Phase 3 trial	Early-stage breast cancer	Anastrozole (1 mg/d)	64 (9)*	100	1102	578	NR	NR	NR	3092	4	16
			Tamoxifen (20 mg/d)	NR	NR	1263	544	NR	NR	NR	3094		
Kaufmann (2007)	Phase 3 trial	Early-stage breast cancer	Anastrozole (1 mg/d)	60.9	30.1	29	NR	52	NR	NR	445	5	17
			Tamoxifen (20–30 mg/d)	60.5	NR	29	NR	22	NR	NR	452		
Coombes (2007)	Phase 3 trial	Early-stage breast cancer	Exemestane (25 mg/d)	NR	55.7	957	526	432	428	454	2320	5	18
			Tamoxifen (20–30 mg/d)	NR	NR	903	522	275	413	393	2338		
Coates (2007)	Phase 3 trial	Early-stage breast cancer	letrozole 2.5mg/d	NR	51	803	NR	489	348	NR	2448	5	19
			Tamoxifen (20 mg/d)	NR	NR	914	NR	331	416	NR	2447		
Jakesz (2005)	Phase 3 trial	Early-stage breast cancer	Anastrozole (1 mg/d)	62·3 (46·0–80·3)	28	537	NR	NR	NR	NR	1120	4	20
			Tamoxifen (20–30 mg/d)	62·0 (41·4–80·0)	NR	560	NR	NR	NR	NR	1117		
Boccardo (2005)	Phase 3 trial	Early-stage breast cancer	Anastrozole (1 mg/d)	63(38–76)	36	NR	4	NR	NR	NR	223	4	5
			Tamoxifen (20 mg/d)	63(43–77)	NR	NR	0	NR	NR	NR	225		
Coombes (2004)	Phase 3 trial	Early-stage breast cancer	Exemestane (25 mg/d)	64.3 (8.1)*	30.6	967	545	124	429	449	2362	4	21
			Tamoxifen (20 mg/d)	64.2 (8.2)*	NR	923	547	85	418	406	2380		
Baum (2003)	Phase 3 trial	Early-stage breast cancer	Anastrozole (1 mg/d)	NR	33	1082	512	NR	NR	NR	3092	4	22
			Tamoxifen (20 mg/d)	NR	NR	1246	491	NR	NR	NR	3093		
Buzdar (1998)	Phase 3 trial	Advanced-stage breast cancer	Anastrozole (1mg/d)	65.6 (10.9)*	31	34	NR	NR	4	NR	262	3	23

☆, date as show with mean(SD); number(percentage);No. of Patients, Number of enrolled patients; NR, Not Reported;

This meta-analysis was performed in accordance with the guidelines of the Preferred Reporting Items for Systematic Reviews and Meta-Analyses (PRISMA) (Supplementary Table 2) [27].

### Overall incidence of MS

Seventeen studies involving 25,062 postmenopausal women examined the association between AIs and MS [5, 11–26]. The index of MS included hot flashes, fatigue, arthralgia, sweating, and insomnia. Hot flashes were observed in 14 of the 17 studies with 7,870 events, and they ranged from 6.52 to 55.89% [11–13, 16–26]. Fatigue was observed in 11 of the 17 studies with 2,331 events, and ranged from 1.34 to 60% [5, 11–14, 16, 18, 21, 22, 25, 26]. Arthralgia was observed in 11 of the 17 studies with 1,672 events, and ranged from 5.25 to 54.29% [11–14, 17–19, 21, 24–26]. Sweating was observed in 4 of the 17 studies with 1,209 events, and ranged from 1.53 to 18.45% [18, 19, 21, 23]. Insomnia was observed in 5 of the 17 studies with 1,640 events, and ranged from 3.36 to 31.12% [13–15, 18, 21].

The overall incidence of hot flashes, fatigue, arthralgia, sweating, and insomnia was 30.47% (95% CI: 25.51%−35.93%, Table 2 and Supplementary Figure 1), 17.16% (95% CI: 14%−20.85%, Table 2 and Supplementary Figure 2), 17.91% (95% CI: 11.29%−27.22%, Table 2 and Supplementary Figure 3), 14.64% (95% CI: 11.46%−18.52%, Table 2 and Supplementary Figure 4), and 16.52% (95% CI: 12.45%−21.6%, Table 2 and Supplementary Figure 5), respectively, according to the random effects model.

**Figure 2:**
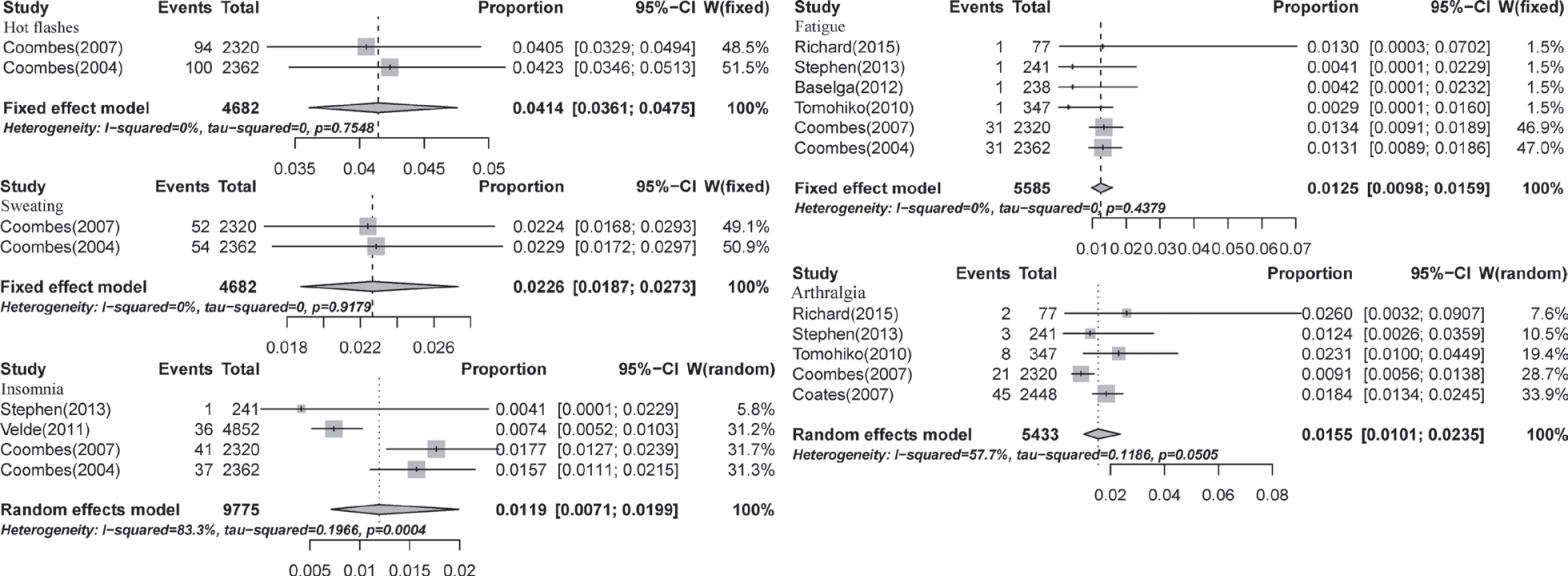
Forest plot for meta-analysis of incidence of all-grade menopausal symptoms in postmenopausal breast cancer patients receiving aromatase inhibitors

High-grade (grades 3 to 5) MS may result in discontinuation of AI treatment and increased morbidity. There were eight studies involving 12,885 patients with high-grade MS [12–15, 18, 19, 21, 26]. The incidence of high-grade hot flashes, fatigue, arthralgia, sweating, and insomnia was 4.14% (95% CI: 3.61%−4.75%, Figure 2), 1.25% (95% CI: 0.98%−1.59%, Figure 2), 1.55% (95% CI: 1.01%−2.35%, Figure 2), 2.26% (95% CI: 1.87%−2.73%, Figure 2), and 1.19% (95% CI: 0.71%−1.99%, Figure 2), respectively, according to the random-effects or fixed-effects models.

### Relative risk of all-gade MS

**Figure 3:**
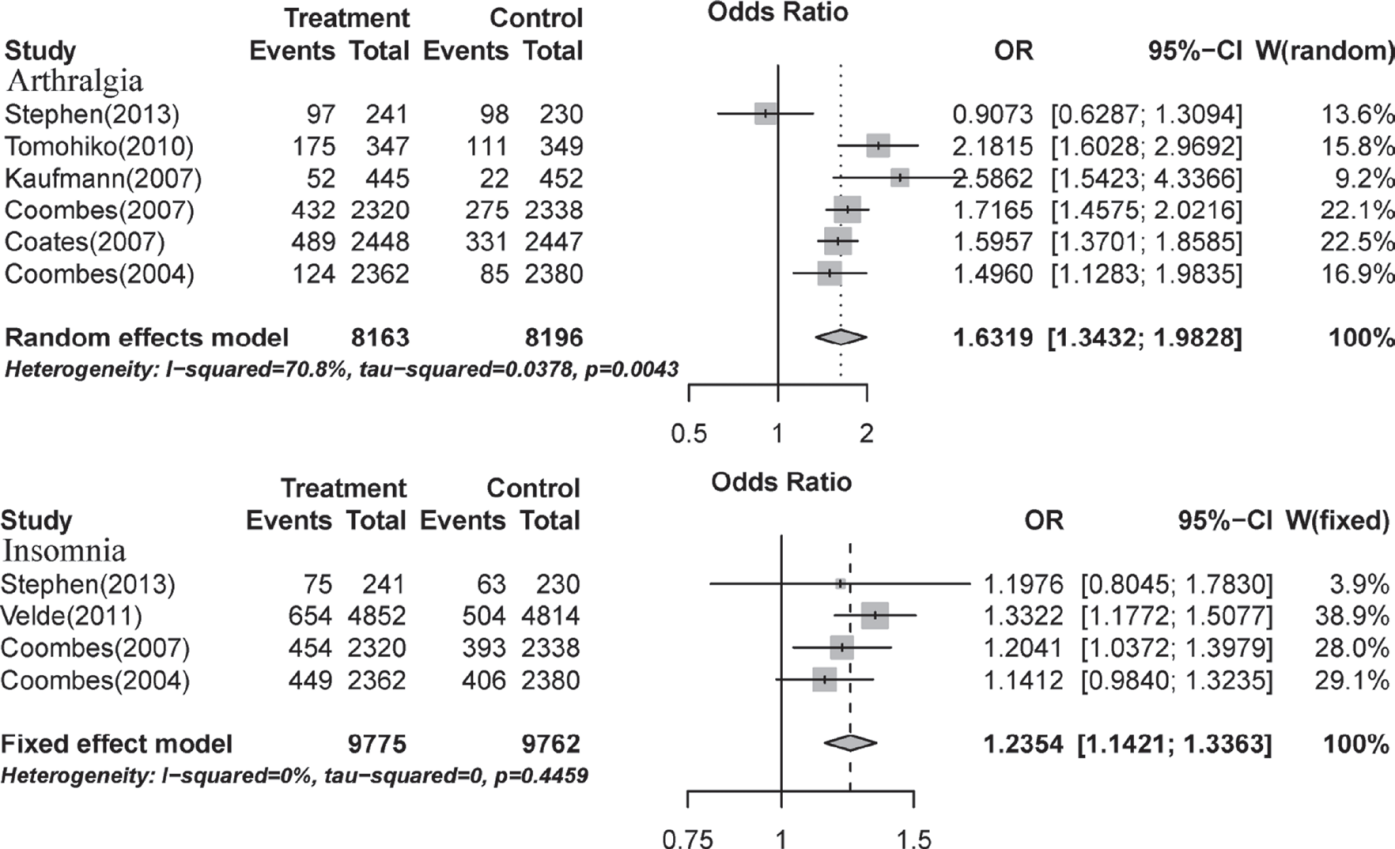
Relative risk of aromatase-inhibitor-associated all-grade arthralgia and insomnia vs. control from included studies with postmenopausal breast cancer

We calculated the odds ratio (OR) of MS between AIs and control groups [5, 13, 15–22, 26]. The pooled ORs of hot flashes, fatigue, arthralgia, sweating, and insomnia were 0.907 (95% CI: 0.809−1.018, *P*-value:0.0966, Table 3 and Supplementary Figure 6), 1.03 (95% CI: 0.97–1.11, *P*-value:0.2552, Table 3 and Supplementary Figure 7), 1.63 (95% CI: 1.34−1.98, *P*-value: < 0.0001, Figure 3), 0.96 (95% CI: 0.81−1.14, *P*-value:0.4284, Table 3 and Supplementary Figure 8), and 1.24 (95% CI: 1.14−1.37, *P*-value:0.0966, Figure 3), respectively, according to random-effects or fixed-effects models.

We also calculated the OR of MS between AIs and tamoxifen groups [5, 15–22, 26]. The pooled ORs of hot flashes, fatigue, arthralgia, sweating, and insomnia were 0.898 (95% CI: 0.797−1.013, *P*-value:0.0797, Supplementary Figure 9), 1.04 (95% CI: 0.97–1.11, *P*-value:0.2449, Supplementary Figure 9), 1.71 (95% CI: 1.55−1.88, *P*-value: < 0.0001, Supplementary Figure 9), 0.96 (95% CI: 0.82−1.14, *P*-value:0.6545, Supplementary Figure 9), and 1.24 (95% CI: 1.14−1.34, *P*-value: < 0.0001, Supplementary Figure 9), respectively, according to random-effects or fixed-effects models.

### Relative risk of high-grade MS

**Figure 4:**
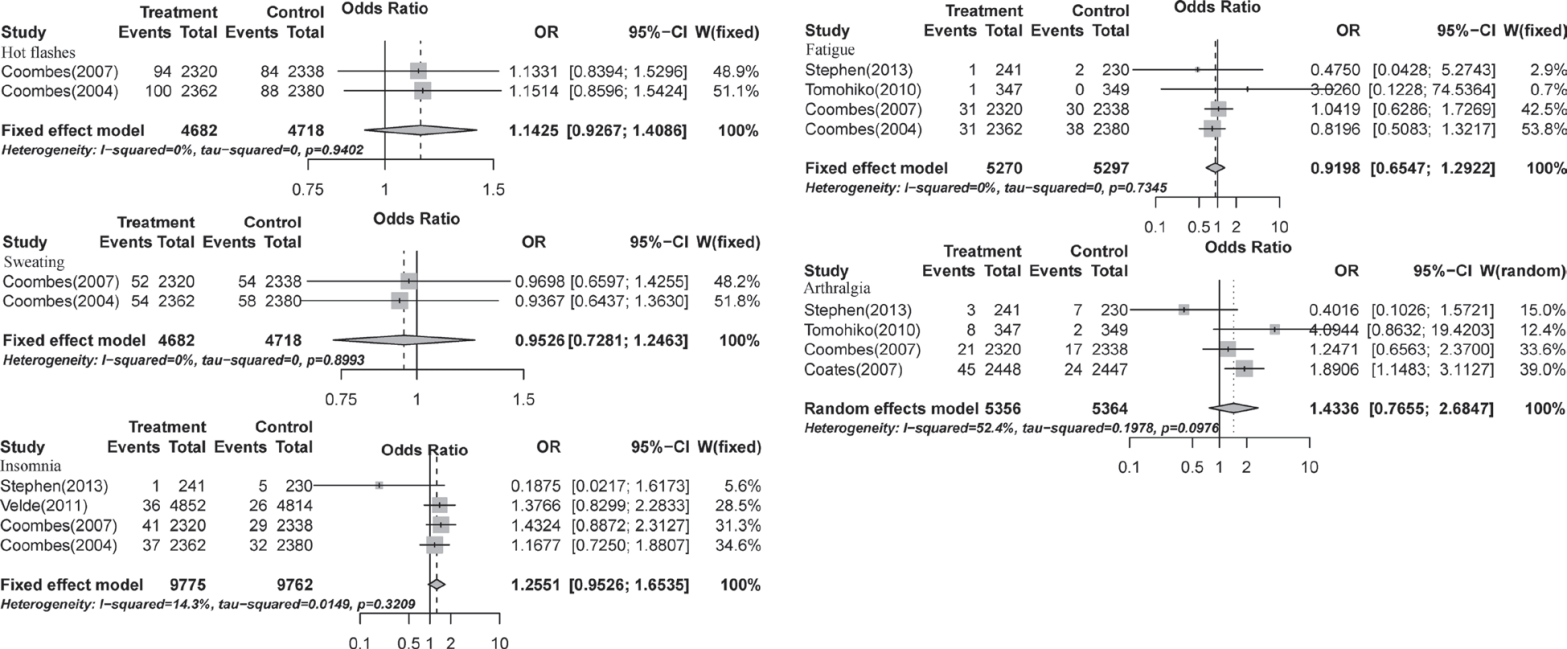
Relative risks of aromatase-inhibitor-associated high-grade menopausal symptoms vs. control from included studies with postmenopausal breast cancer

High-grade MS is an important clinical indicator of AI safety. We analyzed the OR for high-grade MS in AIs and control groups in six RCTs that involved 25,128 patients [13, 15, 18, 19, 21, 26]. The main MS included hot flashes, fatigue, arthralgia, sweating, and insomnia. The pooled ORs of hot flashes, fatigue, arthralgia, sweating, and insomnia were 1.14 (95% CI: 0.93−1.41, *P*-value: 0.2124, Figure 4), 0.92 (95% CI: 0.65−1.29, *P*-value: 0.6245, Figure 4), 1.43 (95% CI: 0.77−2.68, *P*-value: 0.2605, Figure 4), 0.95 (95% CI: 0.73−1.25, *P*-value: 0.7234, Figure 4), and 1.26 (95% CI: 0.95−1.65, *P*-value: 0.1347, Figure 4), respectively, according to random-effects or fixed-effects models.

The analysis was also performed on high-grade MS between AIs and tamoxifen groups [15, 18, 19, 21, 26]. The main MS included hot flashes, fatigue, arthralgia, sweating, and insomnia. The pooled ORs of hot flashes, fatigue, arthralgia, sweating, and insomnia were 1.14 (95% CI: 0.93−1.41, *P*-value: 0.2124, Supplementary Figure 10), 0.93 (95% CI: 0.66−1.32, *P*-value: 0.6936, Supplementary Figure 10), 1.74 (95% CI: 1.19−2.53, *P*-value: 0.0042, Supplementary Figure 10), 0.95 (95% CI: 0.73−1.25, *P*-value: 0.7234, Supplementary Figure 10), and 1.32 (95% CI: 1.00−1.75, *P*-value: 0.0535, Supplementary Figure 10), respectively, according to random-effects or fixed-effects models.

### Incidence of MS in patients with advanced-stage vs. early-stage breast cancer

To determine whether the incidence of MS was associated with breast cancer stage, we analyzed the occurrence of MS in advanced-stage and early-stage breast cancer patients. To calculate the relative risk (RR), we used the comparison of two estimated quantities [28]. The all-grade incidence of arthralgia was significantly decreased in patients with early-stage breast cancer compared with advanced-stage breast cancer (RR = 0.34, 95% CI: 0.16−0.75), but the all-grade incidence of sweating was significantly increased (RR = 11.02, 95% CI: 4.11−29.57). No difference was detected in the all-grade incidence of hot flashes (RR = 1.72, 95% CI: 0.95−3.09), fatigue (RR = 1.52, 95% CI: 0.30–7.59), and insomnia (RR = 1.51, 95% CI: 0.19–11.83).

No statistical difference was found in the high-incidence of fatigue (RR = 2.16 [95% CI 0.69–6.80]), arthralgia (RR = 0.78 [95% CI: 0.26–2.38]), and insomnia (RR = 3.10 [95% CI 0.42–23.04]) between patients with advanced-stage and early-stage cancers.

### Publication bias

**Figure 5:**
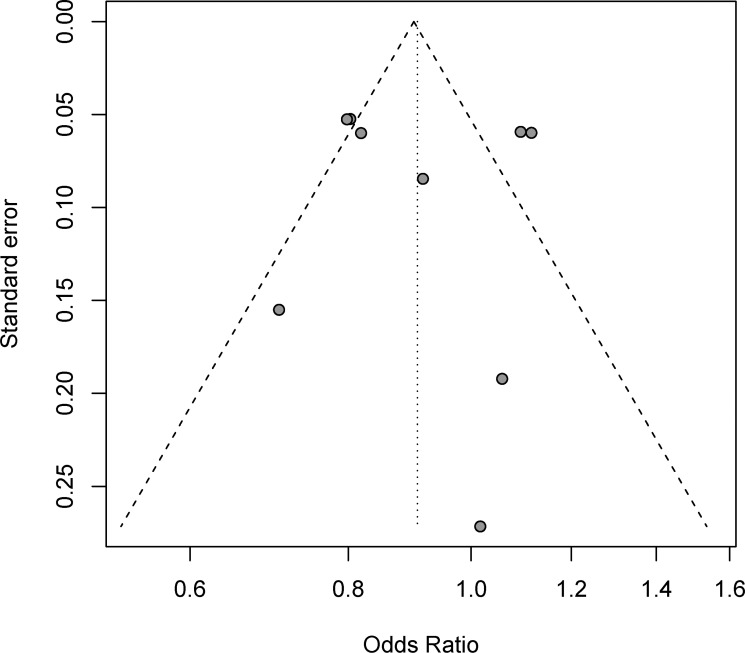
Funnel plots of studies reporting risk ratio for all-grade hot flashes in postmenopausal breast cancer patients receiving aromatase inhibitors and control

No evidence of publication bias was found for the OR of MS of hot flashes in our meta-analysis by funnel plots (Figure 5), Egger's test (*P* = 0.891 > 0.05, 95% CI: −2.81, 3.17), or Begg's test (*Z* = 0.12 < 1.96, *P* = 0.903 > 0.05). Heterogeneity of different rates of occurrence in the various clinical trials was statistically significant, and data of MS were analyzed using a random-effects model.

## DISCUSSION

Our study indicates that the use of AIs in breast cancer patients is associated with an increased risk of menopausal symptoms (MS). Patients on AI therapy had a high overall incidence of all-grade MS, including hot flashes (30.47%), fatigue (17.16%), arthralgia (17.91%), sweating (14.64%), and insomnia (16.52%). They also had a high overall incidence of high-grade MS, including hot flashes (4.14%), fatigue (1.25%), arthralgia (1.55%), sweating (2.26%), and insomnia (1.19%). Aggressive and adequate management of moderate MS is important for cancer patients, because the negative effect of endocrine therapy on quality of life is a major concern [29, 30].

Our data are consistent with previous studies demonstrating the prevalence of vasomotor symptoms, such as sweating and hot flashes, and fatigue, arthralgia, and insomnia in breast cancer patients [29, 31]. In addition, we have found that the AI therapy is associated with an increased incidence of all-grade arthralgia and insomnia, but not with hot flashes, fatigue, or sweating. The ORs of high-grade incidences of hot flashes, fatigue, arthralgia, sweating, and insomnia were 1.14, 0.92, 1.43, 0.95, and 1.26, respectively. Because of the increased risk of arthralgia and insomnia, it is necessary for physicians and oncologists to be aware of the increased risk of MS associated with AIs and treat them appropriately.

The risk of MS may vary depending on the tumor stage. Indeed, our results indicate that the incidence of arthralgia in early-stage breast cancer patients is lower than in advanced-stage breast cancer patients, whereas the incidence of sweating in early stages is higher than in other stages. The different occurrence of arthralgia and sweating in different tumor-stages may be due to the development of the disease itself and/or medications. The patients need to know that there are different MS depending on the disease stages.

The MS during natural or medically induced menopause are associated with the rapid decline of estrogen levels [32, 33]. Premenopausal and reproductive-age women usually do not suffer from these symptoms [34]. In contrast, breast cancer patients often suffer from MS because of endocrine therapy or chemotherapy [35–37]. Therefore, having MS following therapy for a breast tumor may indicate decreased circulating estrogen levels and favorable prognosis [38, 39]. This hypothesis is supported by our data indicating that breast cancer patients in the AI group have increased occurrence of MS compared to patients in the control group.

The management of AI-associated MS is still controversial and difficult. The role of AIs in MS symptoms may vary with the patient's age. The MS risk of women who just crossed the menopause may be higher compared to older patients. Bone-density screening should be performed in all postmenopausal breast cancer patients, since AIs can accelerate bone loss [40]. In general, hormone replacement therapy (HRT) should be avoided after an early diagnosis of breast cancer, since clinical trials have reported that it increases the risk of breast cancer recurrence [41, 42]. Since AI therapy induces MS in breast cancer patients, it may be difficult for them to distinguish whether their symptoms are induced by the drug treatment or by the disease itself. To distinguish between these two possibilities, it is sometimes necessary to suspend the drug treatment for two or more weeks. Once the majority of MS disappear, then reassessing the case or trying a different drug may be necessary [40]. Our finding is that high-grade MS of hot flashes, fatigue, arthralgia, sweating, and insomnia are not significantly different in patients receiving the AI therapy compared to other therapies, but the risk of all-grade arthralgia and insomnia is significantly increased in patients with AI treatment.

As with any meta-analysis, this study has some limitations. First, the included studies were performed at various international institutions by different researchers, and there may be a bias in the reporting of adverse events. Different methods of symptoms and risk observation were recorded in different studies. In particular, the incidence rate of MS is understated or missing in some clinical trials. Second, the patients’ baseline MS were not reported in the studies, which might have led to an overestimation of the risk of AI-associated MS. Third, there might have been potentially important differences among the studies, including different tumor types, AI dosages and administration schedules, periods of study, and study investigators. In addition, three different AIs have been used in the included studies; while exemestane is a steroidal AI, anastrozole and letrozole are non-steroidal AIs. All these factors increase the clinical heterogeneity among the included trials, making the interpretation of a meta-analysis challenging. Additionally, since RCTs have strict inclusion and exclusion criteria, the results of this meta-analysis may not represent those found in patients [43, 44].

Together, our study shows that AIs are associated with a significant risk of developing all-grade MS of arthralgia and insomnia, and that the risk of arthralgia and sweating depends on the tumor stage. Effective early detection and management of MS should lead to safer use of AIs in breast cancer patients.

## MATERIALS AND METHODS

### Search strategy and study selection

This study was performed according to the Preferred Reporting Items for Systematic Reviews and Meta-Analyses (PRISMA) guidelines. The databases of EMBASE, PubMed, and the Cochrane Library were searched through August 3, 2016, with English language restriction. The computer search terms included the following free text and MeSH terms: “breast neoplasms,” “breast cancer,” “breast tumor,” “mammary cancer,” “aromatase inhibitors,” “anastrozole,” “letrozole,” and “clinical trial.” The details of the search strategy are summarized in Supplementary Table 1. Two independent investigators selected the eligible studies according to the selection criteria, and discrepancies were resolved by consensus. Studies were suitable if they met the following criteria: (1) Randomized clinical trials (RCTs) in postmenopausal patients with breast cancer. (2) Patients treated with aromatase inhibitors, including anastrozole, letrozole, or exemestane. (3) Data regarding end points for MS, including hot flashes, fatigue, arthralgia, sweating, and insomnia [45, 46]. These clinical end points were obtained according to the Common Terminology Criteria for Adverse Events (CTCAE) of the National Cancer Institute (https://ctep.cancer.gov/protocoldevelopment/electronic_applications/ctc.htm#ctc_archive).

### Data extraction and quality assessment

Index of the MS included hot flashes, fatigue, arthralgia, sweating, and insomnia. Data were extracted from the safety profile of each RCT. Two independent investigators extracted data that included patient characteristics, regional distribution, treatment measures, follow-up time, and outcome data. Quality assessment was performed by the methodologies of Jadad criteria [47], and studies with scores of ≥ 3 were classified as high quality.

### Data analysis

The principal summary measures were incidence, odds ratio (OR), and corresponding 95% CI. To calculate incidence, the number of patients with MS and the number of patients receiving aromatase inhibitors were extracted. The proportion of patients with MS and 95% CI were calculated in each study. The OR of MS was calculated only with those assigned to the control group in the same trial. A statistical test with a *P value* less than 0.05 was considered significant. We used the Peto method to calculate the OR and 95% CI because this method provides the best confidence interval coverage, and it is more powerful and less biased when calculating low event rates [48]. Heterogeneity among clinical trials was assessed using the Q statistic and I2 tests.[49, 50] To calculate the pooled incidence, an inverse variance statistical method was used. Heterogeneity was considered statistically significant when *P* < 0.1 or I2 > 40%. If heterogeneity existed, the data were analyzed using a random-effects model; if heterogeneity did not exist, a fixed-effects model was used. The presence of publication bias was evaluated using the funnel plot, Begg's test, and Egger's test [51, 52]. All data analyses were performed using R software version 3.0.3 (R foundation for statistical computing, http://www.r-project.org).

## SUPPLEMENTARY MATERIALS FIGURES AND TABLES






