# Supplementary material for: Incidence of menopausal symptoms in postmenopausal breast cancer patients treated with aromatase inhibitors
Source: Oncotarget. 2017 Apr 18;8(25):40558–67. doi: 10.18632/oncotarget.17194 (PMC5522209; doi:10.18632/oncotarget.17194)
Supplement: Supplementary file 1 [file oncotarget-08-40558-s001.pdf]

Incidence of menopausal symptoms in postmenopausal breast cancer patients treated with aromatase inhibitors

Supplementary Materials

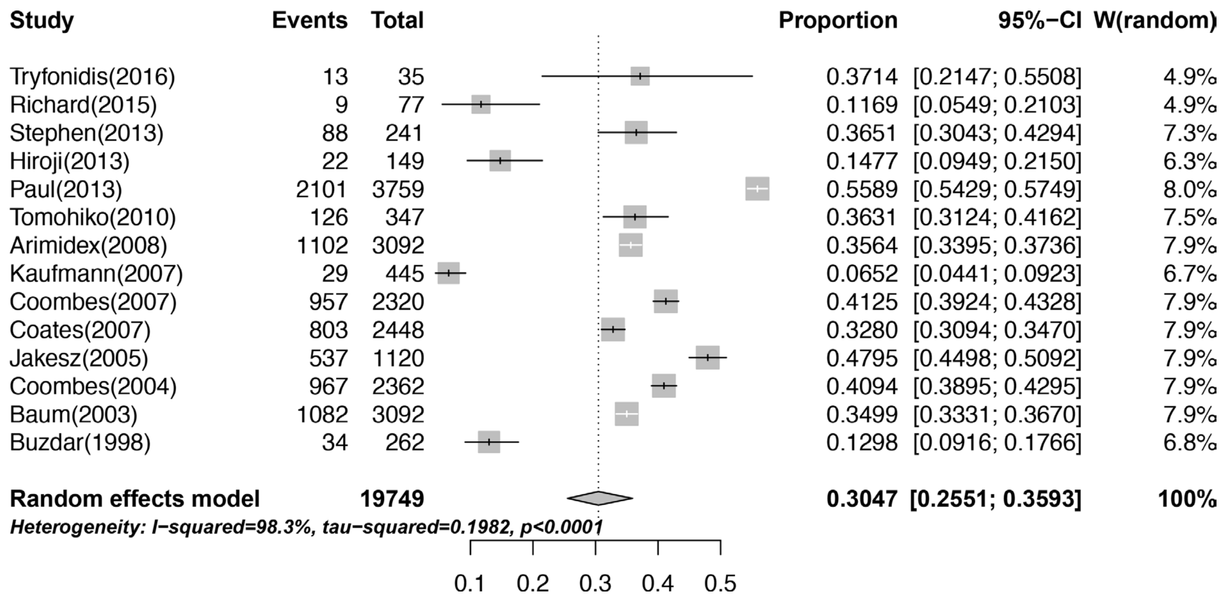

Supplementary Figure 1: Forest plot for meta-analysis of incidence of all-grade hot flashes in postmenopausal breast cancer patients receiving aromatase inhibitors.

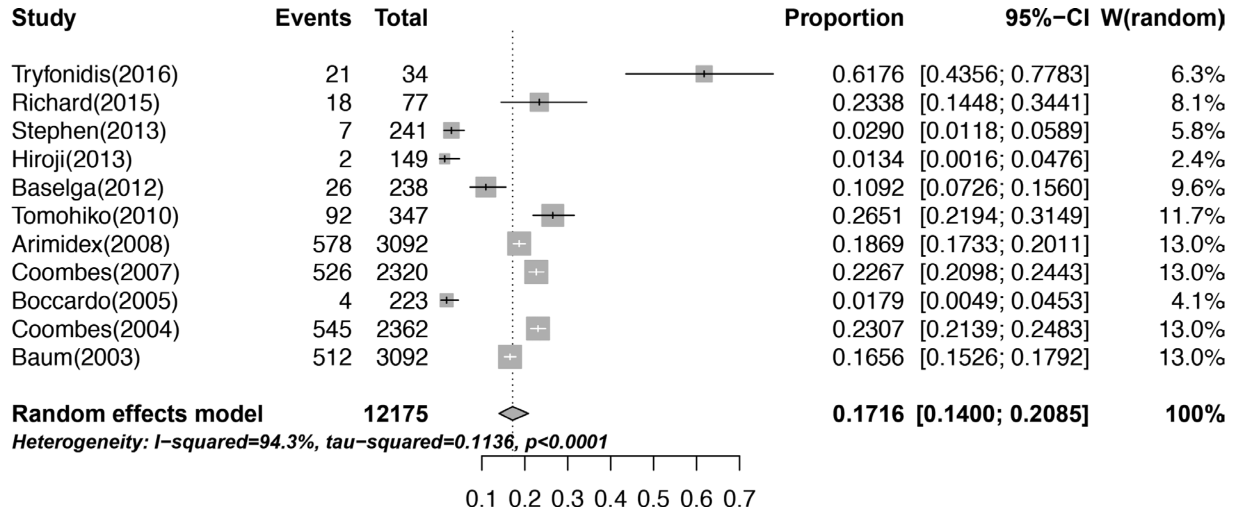

Supplementary Figure 2: Forest plot for meta-analysis of incidence of all-grade fatigue in postmenopausal breast cancer patients receiving aromatase inhibitors.

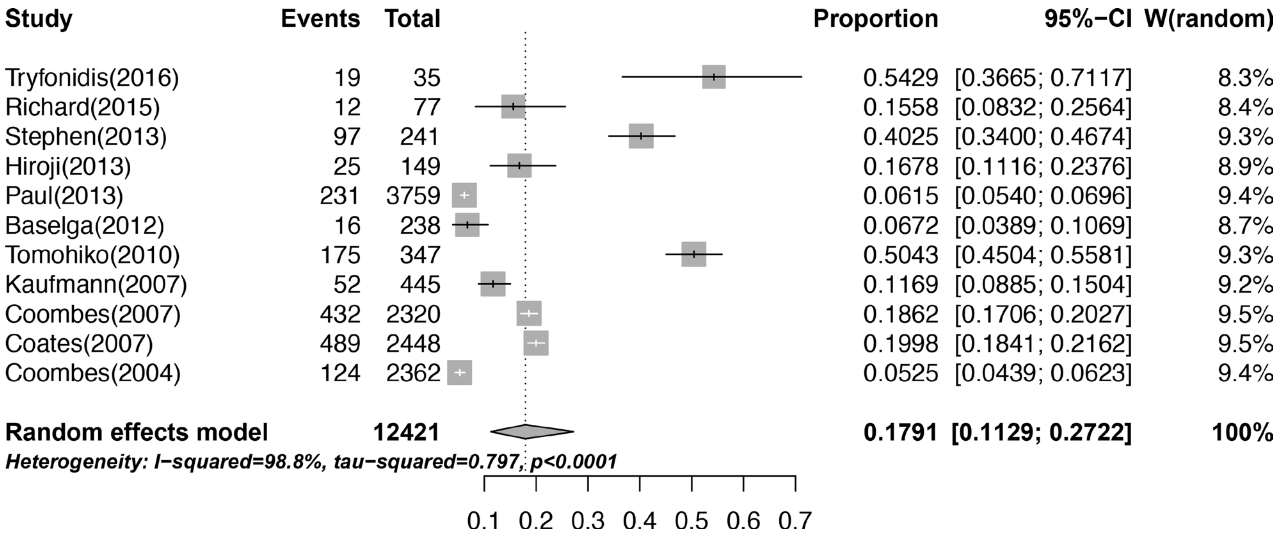

Supplementary Figure 3: Forest plot for meta-analysis of incidence of all-grade arthralgia in postmenopausal breast cancer patients receiving aromatase inhibitors.

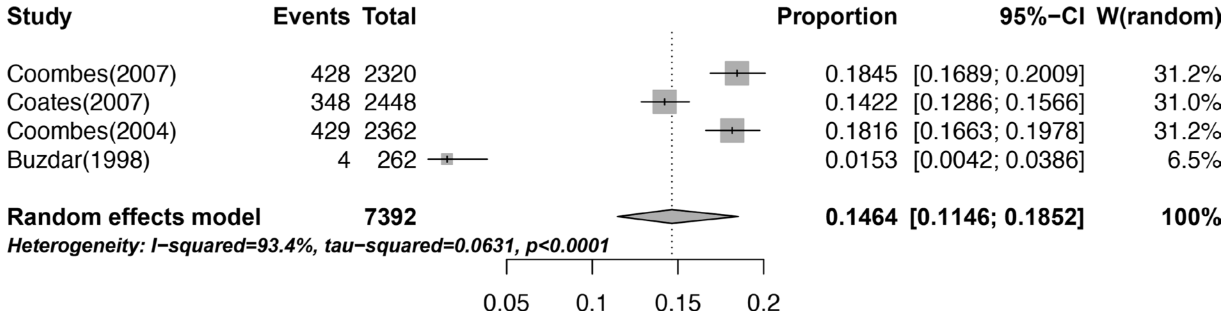

Supplementary Figure 4: Forest plot for meta-analysis of incidence of all-grade sweating in postmenopausal breast cancer patients receiving aromatase inhibitors.

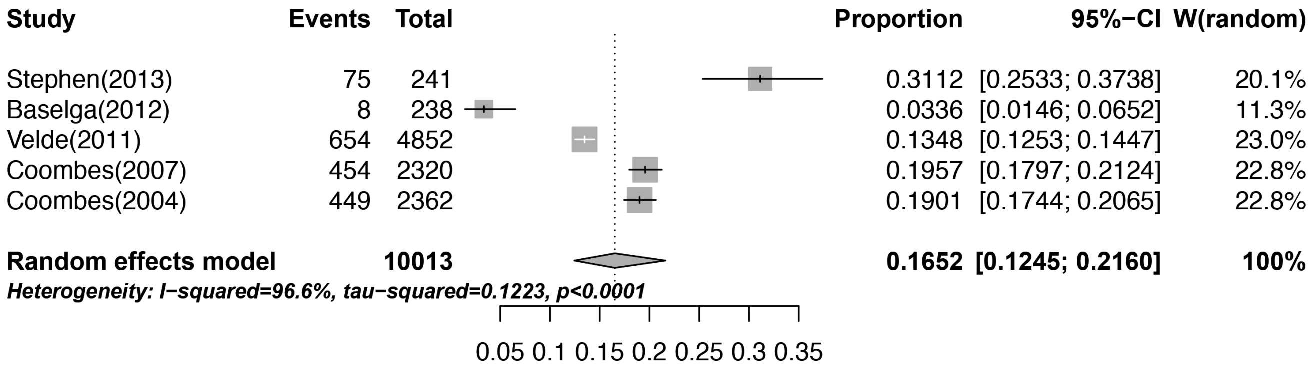

Supplementary Figure 5: Forest plot for meta-analysis of incidence of all-grade insomnia in postmenopausal breast cancer patients receiving aromatase inhibitors.

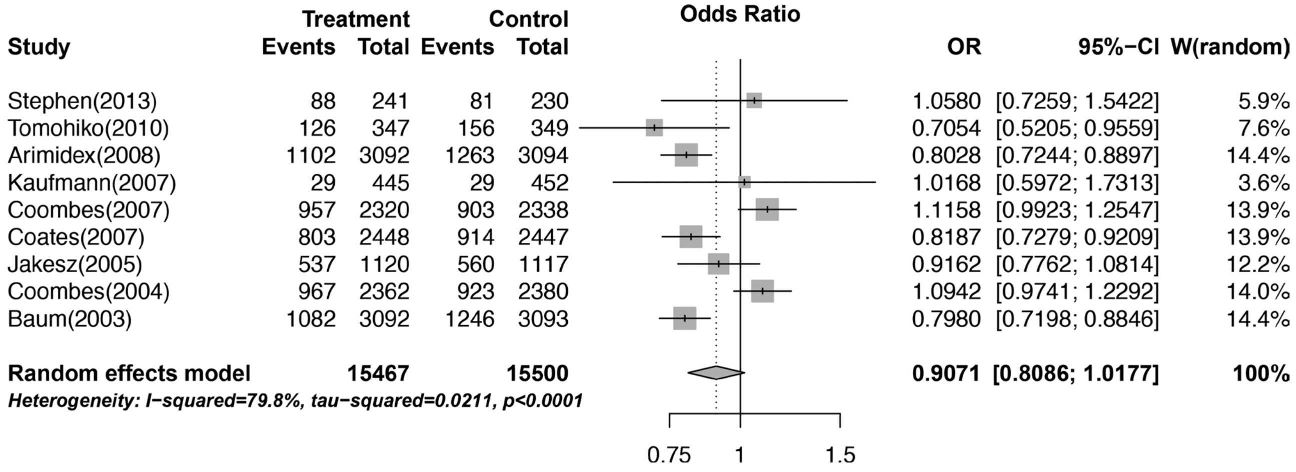

Supplementary Figure 6: Relative risk of aromatase-inhibitor-associated all-grade hot flashes vs. control from included studies with postmenopausal breast cancer.

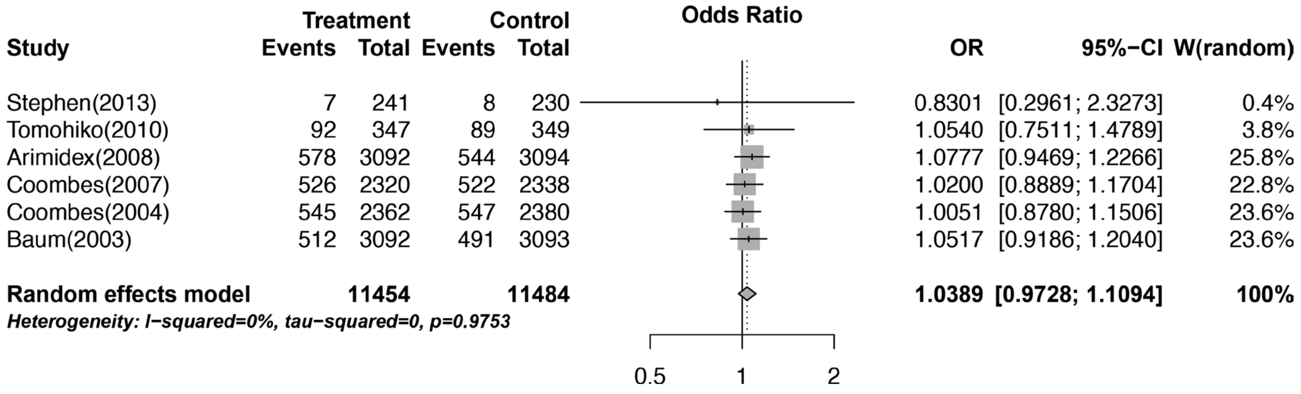

Supplementary Figure 7: Relative risk of aromatase-inhibitor-associated all-grade fatigue vs. control from included studies with postmenopausal breast cancer.

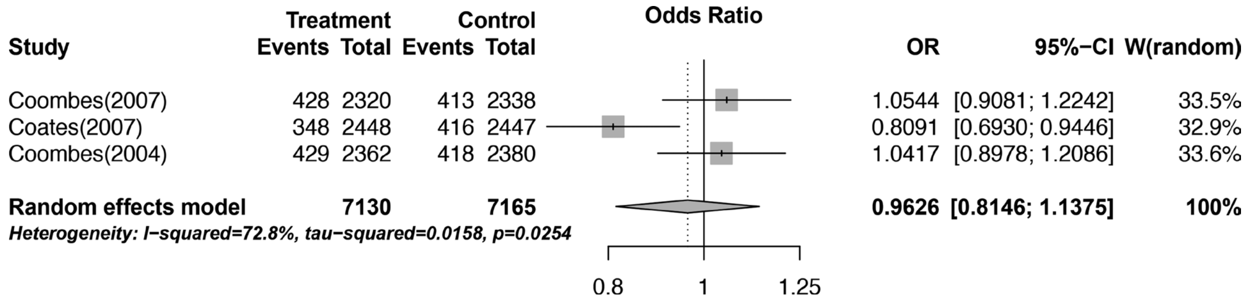

Supplementary Figure 8: Relative risk of aromatase-inhibitor-associated all-grade sweating vs. control from included studies with postmenopausal breast cancer.

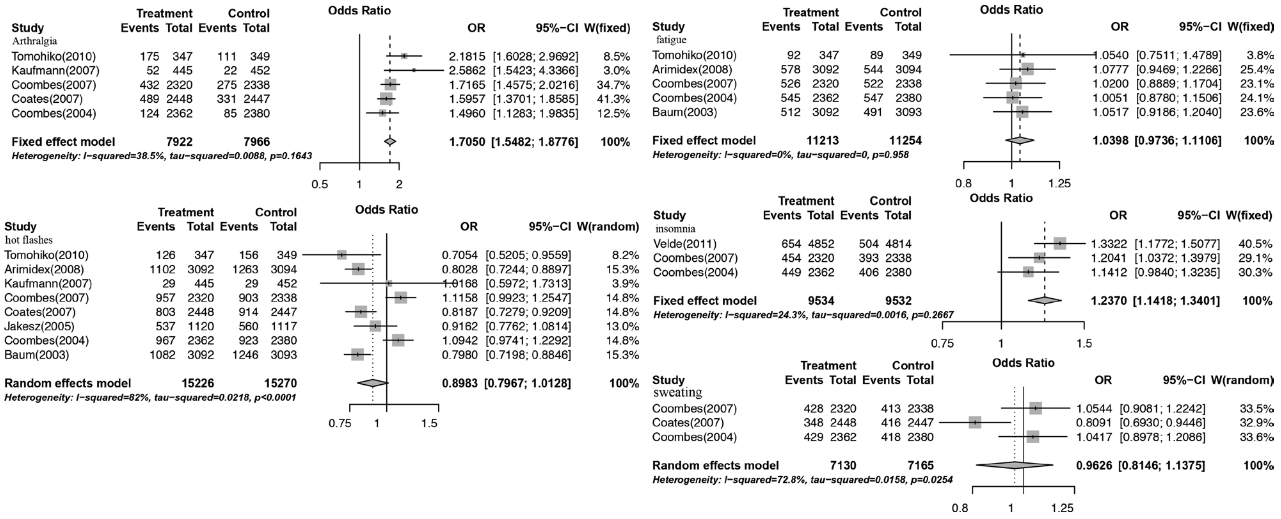

Supplementary Figure 9: Relative risk of aromatase-inhibitor-associated all-grade menopausal symptoms vs. tamoxifen from included studies with postmenopausal breast cancer.

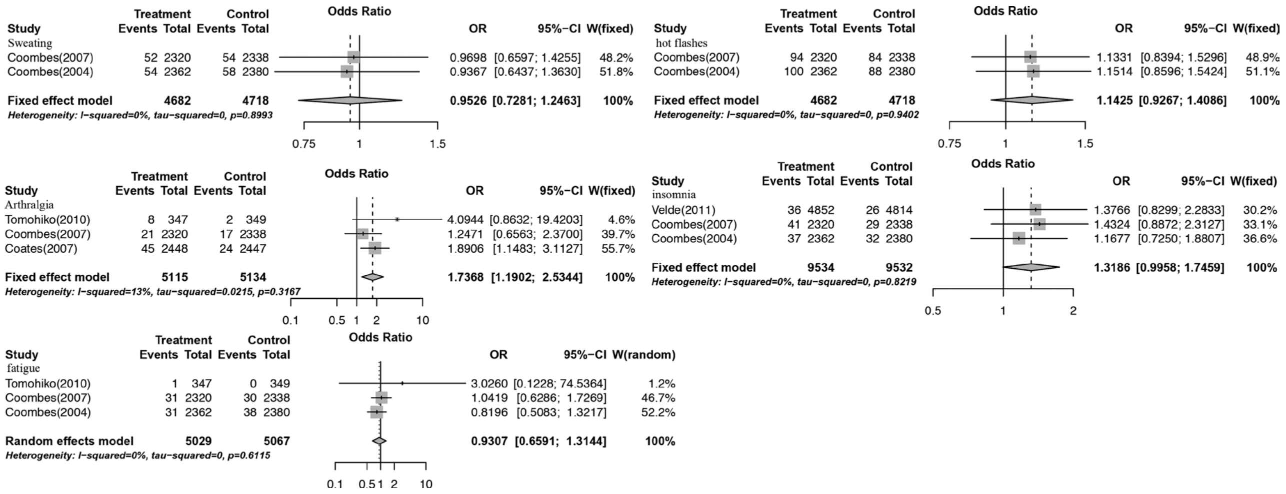

Supplementary Figure 10: Relative risk of aromatase-inhibitor-associated high-grade menopausal symptoms vs. tamoxifen from included studies with postmenopausal breast cancer.

Supplementary Table 1: Search strategy for systematic review of Menopausal symptoms in postmenopausal breast cancer patients receiving aromatase inhibitors or control. See_ Supplementary_Table_1

Supplementary Table 2: PRISMA 2009 checklist for systematic review on Menopausal symptoms in postmenopausal breast cancer patients receiving aromatase inhibitors or control. See_ Supplementary_Table_2

Supplementary Table 3: World distribution of randomized clinical trials included in the meta-analysis

Author (Publication Date)	Journal	Location
Tryfonidis(2016)	European Journal of Cancer	Europe
Richard(2015)	Lancet Oncology	North America ,Europe, Asia-Pacific,Africa
Stephen(2013)	Lancet Oncology	Europe and Asia-Pacific
Hiroji(2013)	Breast Cancer Res Treat	Asia-Pacific
Paul(2013)	Journal of Clinical Oncology	North America
Baselga(2012)	The New England and Journal of Medicine	North America ,Europe, Asia-Pacific
Velde(2011)	Lancet	North America ,Europe, Asia-Pacific
Tomohiko(2010)	Breast Cancer Res Treat	Asia-Pacific
Arimidex(2008)	Lancet Oncology	Europe
Kaufmann(2007)	Journal of Clinical Oncology	Europe
Coombes(2007)	Lancet	North America ,Europe
Coates(2007)	Journal of Clinical Oncology	North America ,Europe, Asia-Pacific
Jakesz(2005)	Lancet	Europe
Boccardo(2005)	Journal of Clinical Oncology	Europe
Coombes(2004)	The New England and Journal of Medicine	Europe
Baum(2003)	Cancer	Europe
Buzdar(1998)	Cancer	North America ,Europe
