# Supplementary material for: Incidence of menopausal symptoms in postmenopausal breast cancer patients treated with aromatase inhibitors
Source: Oncotarget. 2017 Apr 18;8(25):40558–67. doi: 10.18632/oncotarget.17194 (PMC5522209; doi:10.18632/oncotarget.17194)
Supplement: Supplementary file 2 [file oncotarget-08-40558-s002.doc]

**Table S1 Search strategy for systematic review of Menopausal symptoms in postmenopausal breast cancer patients receiving aromatase inhibitors or control**

**TABLE S1.1 Pubmed** via NLM,OCT 07, 2016.

|  | Search terms: *Aromatase inhibitors for breast cancer* | Items found |
| --- | --- | --- |
| Population: persons with breast cancer | | |
|  | Search ((((((((((((breast neoplasms[Title/Abstract]) OR breast cancer[Title/Abstract]) OR breast neoplasm[Title/Abstract]) OR breast tumor[Title/Abstract]) OR breast tumour[Title/Abstract]) OR breast tumors[Title/Abstract]) OR breast tumours[Title/Abstract]) OR mammary cancer[Title/Abstract]) OR mammary tumor[Title/Abstract]) OR mammary tumors[Title/Abstract]) OR mammary tumour[Title/Abstract]) OR mammary tumours[Title/Abstract]) OR "Breast Neoplasms"[Mesh] | 304962 |
| Intervention: Aromatase Inhibitors | | |
|  | Search ((((((((aromatase inhibitors[Title/Abstract]) OR aromatase inhibitor[Title/Abstract]) OR exemestane[Title/Abstract]) OR letrozole[Title/Abstract]) OR anastrozole[Title/Abstract]) OR "anastrozole" [Supplementary Concept]) OR "letrozole" [Supplementary Concept]) OR "exemestane" [Supplementary Concept]) OR "Aromatase Inhibitors"[Mesh] | 8801 |
| Study types: | | |
|  | ("Clinical Trial" [Publication Type] OR "Clinical Trials as Topic"[Mesh] OR "case control studies"[MeSH Terms] OR "cohort studies"[MeSH Terms] OR "observational"[Title/Abstract] OR "observation"[Title/Abstract] OR "random"[Title/Abstract] OR "randomly"[Title/Abstract] OR "randomized"[Title/Abstract] OR "randomised"[Title/Abstract] OR "control"[Title/Abstract] OR "controlled"[Title/Abstract] OR "clinical trial"[Title/Abstract] OR "case control"[Title/Abstract] OR "longitudinal"[Title/Abstract] OR "prospective"[Title/Abstract] OR "long term"[title/abstract] OR "longterm"[title/abstract] OR "follow up"[Title/Abstract] OR "followup"[Title/Abstract] OR systematic[sb] OR "Meta-Analysis"[Publication Type]) | 3451821 |
| Combined sets | | |
|  | 1 AND 2 AND 3 | 2202 |
| Limits: date | | |
|  | 4 AND "english"[Language] | 2059 |

[MeSH] = Term from the Medline controlled vocabulary, including terms found below this term in the MeSH hierarchy; [TI] = Title;

* = Truncation; “ “ = Citation Marks; searches for an exact phrase

**TABLE S1.2 Embase via Embase.com, OCT 07, 2016.**

|  | Search terms: *Aromatase inhibitors for early stage breast cancer* | Items found |
| --- | --- | --- |
| Population: persons with breast cancer | | |
|  | 'breast tumor':ab,ti or 'breast neoplasm':ab,ti or 'breast neoplasms':ab,ti or 'breast cancer':ab,ti or 'breast tumor':ab,ti or 'breast tumour':ab,ti or 'breast tumours':ab,ti or 'breast tumors':ab,ti or 'mammary cancer':ab,ti or 'mammary tumor':ab,ti or 'mammary tumors':ab,ti or 'mammary tumours':ab,ti or 'mammary tumour':ab,ti or 'breast tumor'/exp or 'breast cancer'/exp | 454243 |
| Intervention: Aromatase Inhibitors | | |
|  | 'aromatase inhibitor':ab,ti OR 'aromatase inhibitors':ab,ti OR 'exemestane':ab,ti OR 'letrozole':ab,ti OR 'anastrozole':ab,ti OR 'anastrozole'/exp OR 'letrozole'/exp OR 'exemestane'/exp OR 'aromatase inhibitor'/exp | 25543 |
| Study types: | | |
|  | 'controlled clinical trial':ab,ti OR 'clinical trial':ab,ti OR 'randomized controlled trial':ab,ti OR 'randomized controlled trial'/exp OR 'clinical trial'/exp OR 'controlled clinical trial'/exp OR 'controlled clinical trial (topic)'/exp OR 'clinical trial (topic)'/exp OR 'randomized controlled trial (topic)'/exp | 1,321,732 |
| Combined sets | | |
|  | 1 AND 2 AND 3 | 6130 |
| Limits: date | |  |

/exp= Includes terms found below this term in the EMTREE hierarchy

/mj = Major Topic

:ab = Abstract; :au = Author; :ti = Article Title; ti:ab = Title or abstract

* = Truncation; “ “ = Citation Marks; searches for an exact phrase

**TABLE S1.3 Cochrane Library via Wiley (CENTRAL), OCT 07, 2016.**

|  | | Search terms: *Aromatase inhibitors for early stage breast cancer* | Items found |
| --- | --- | --- | --- |
| Population: persons with breast cancer | | | |
| 1 | breast neoplasm:ti,ab,kw (Word variations have been searched) or breast cancer:ti,ab,kw (Word variations have been searched) or breast tumor:ti,ab,kw (Word variations have been searched) or mammary cancer:ti,ab,kw (Word variations have been searched) or mammary tumour:ti,ab,kw (Word variations have been searched) or MeSH descriptor: [Breast Neoplasms] explode all trees | | 21797 |
| Intervention: Aromatase Inhibitors | | | |
| 2 | aromatase inhibitor:ti,ab,kw (Word variations have been searched) or anastrozole:ti,ab,kw (Word variations have been searched) or exemestane:ti,ab,kw (Word variations have been searched) or MeSH descriptor: [Aromatase Inhibitors] explode all trees or letrozole:ti,ab,kw (Word variations have been searched) | | 2061 |
| Combined sets: | | | |
| 4 | 1 AND 2 AND 3 | | 1551 |
| Limits: date | | | |
| 5 | 4 AND "Trials" | | 1448 |

[MeSH] = Term from the Medline controlled vocabulary, including terms found below this term in the MeSH hierarchy

[TI] = Title; [TIAB] = Title or abstract; [AU] = Author;

[TW] = Text Word

* = Truncation; “ “ = Citation Marks; searches for an exact phrase
